# Serum levels of microRNA-371a-3p are not elevated in testicular tumours of non-germ cell origin

**DOI:** 10.1007/s00432-020-03429-x

**Published:** 2020-11-16

**Authors:** Gazanfer Belge, Francesca Grobelny, Arlo Radtke, Jacqueline Bodes, Cord Matthies, Christian Wülfing, Klaus-Peter Dieckmann

**Affiliations:** 1 Faculty of Biology and Chemistry, University of Bremen, Bremen, Germany; 2 Department of Urology, Bundeswehrkrankenhaus Hamburg, Hamburg, Germany; 3 Department of Urology, Asklepios Klinik Altona, Paul-Ehrlich-Str. 1, 22763 Hamburg, Germany

**Keywords:** Biomarker, miR-371a-3p, Non-germ cell tumour, Malignant lymphoma, Leydig cell tumour, Sertoli cell tumour

## Abstract

**Purpose:**

Serum levels of microRNA-371a-3p (M371) have been shown to be a highly sensitive and specific biomarker for testicular germ cell tumours (TGCT). Little information exists on the expression of this marker in testicular neoplasms deriving from the gonadal stroma or other structures of the gonad. This study presents an expression analysis of the novel TGCT-biomarker M371 in a large cohort of testicular non-germ cell tumours.

**Methods:**

The M371 expression was measured by quantitative real time PCR in serum of 99 patients with testicular tumours of non-germ cell origin, thereof 30 patients with malignant testicular lymphomas and 61 patients with gonadal stroma tumours such as Leydig cell tumours, Sertoli cell tumours and 8 cases with miscellaneous benign testicular tumours. Their M371 levels were compared to those of 20 patients with TGCT and to 37 tumour-free male controls.

**Results:**

The median expression levels of benign testicular tumours and testicular lymphoma are close to zero, thus, identical with those of controls and significantly lower than those of TGCT. In summary, this study provides further evidence for the notion that M371 is exclusively expressed by germ cell tumours and not by testicular neoplasms of the non-germ cell subtypes.

**Conclusion:**

Clinically, the test might be of value in preoperative characterization of benign testicular tumours eligible for conservative surgery.

## Introduction

Testicular germ cell tumours (TGCTs) represent a paradigm of a curable malignancy (Rajpert-De Meyts et al. 2016). Clinical management of TGCTs is extensively guided by imaging technologies e.g. computed tomography or magnetic resonance imaging and by serum levels of tumour markers alpha fetoprotein (AFP), beta Human chorionic gonadotropin (bHCG) and lactate dehydrogenase (LDH) (Albers et al. 2015; Honecker et al. 2018; Lembeck et al. 2020). Unfortunately, the utility of the classical tumour markers is compromised by their low sensitivity. Recently, serum levels of microRNA-371a-3p (so called M371 test) have been shown to greatly outperform the classical markers with a sensitivity of 90% and a specificity of around 94% (Almstrup et al. 2020; Dieckmann et al. 2019; Lobo et al. 2019; Terbuch et al. 2018). Histologically, 90% of all testicular tumours are of germ cell origin. The remainder comprises of an extraordinarily wide variety of rare neoplasms (Idrees et al. 2017; Mooney and Kao 2018). The most frequent subgroup of non-germ cell tumours develop from the gonadal stroma such as Leydig cell tumours, Sertoli cell tumours and granulosa cell tumours including cases with mixed forms and unspecified stromal tumours. More than 90% of these neoplasms are of benign nature (Ruf et al. 2020). The other major subgroup of testicular non-germ cell tumours comprises of so-called haematolymphoid tumours with diffuse large B-Cell lymphoma representing the most frequent subtype followed by follicular lymphoma and rare hematological malignancies such as plasmacytoma and others. The WHO also lists a group of miscellaneous neoplasms with excessively rare incidences that mainly comprise of ovarian epithelial-type tumours with testicular thecoma representing one of these. To complete the list of non-germ cell neoplasms of the testis, the excessively rare and highly malignant carcinoma of the rete testis must be noted as well as metastases of other malignancies to the testicle (Dieckmann et al. 1988). The clinical management of non-germ cell tumours of the testis is hampered by the unpredictable natural course of many of these neoplasms and by the lack of a serum biomarker for assessing the state of the disease (Banerji et al. 2016; Fankhauser et al. 2019; Kern et al. 2020). It was shown that M371 is not or only very little expressed by extra-testicular malignancies (Spiekermann et al. 2015), but there are currently limited data regarding the expression of this novel marker in testicular neoplasms of non-germ cell origin (Regouc et al. 2020). The present study analyses serum levels of M371 in a large patient sample of non-germ cell testicular tumours with several histological subgroups and compares the findings with the levels in patients with TGCT.

## Materials and methods

### Patients for serum investigations

A total of 99 patients with non-germ cell tumours of the testis underwent cubital vein blood aspiration for analysis. The patient population involved the following histological subgroups: Leydig cell tumour (*n* = 40, median age 43.0 years), Sertoli cell tumours (*n* = 18, median age 40.5 years), Granulosa cell tumour (*n* = 2, median age 27.0 years) and one unspecified gonadal stroma tumour (age 21 years) as well as testicular adenomatoid tumours (*n* = 6, median age 34.0 years), testicular hemangioma (*n* = 2, median age 72.5 years) and malignant testicular lymphoma (*n* = 30, median age 69.5 years) (Table 1). The majority of patients were recruited from Hamburg-based hospitals: Albertinen-Krankenhaus, Asklepios Klinik Altona, and Bundeswehr Krankenhaus during 2015–2019. A few additional cases were originally recruited by other institutions for a multicentric study on GCT patients but excluded from that evaluation because of their non-GCT histology (Dieckmann et al. 2019). All serum samples were collected preoperatively. Twenty patients with testicular germ cell tumour (14 seminoma, 6 nonseminoma; median age 34.5 years) (Table 2), and 37 tumour-free males (median age 40.0 years) served as controls (Table 3).

**Table 1:** Clinical data and relative M371 expression in serum of patients with testicular non-germ cell tumours

Patient ID	Age (years)	Dignity	Histology	RQ M371
1	30	Benign	Leydig cell tumor	0.00
2	47	Benign	Leydig cell tumor	0.00
3	22	Benign	Leydig cell tumor	0.00
4	44	Benign	Leydig cell tumor	0.00
5	52	Benign	Leydig cell tumor	0.00
6	64	Benign	Leydig cell tumor	0.00
7	44	Benign	Leydig cell tumor	0.00
8	39	Benign	Leydig cell tumor	0.00
9	37	Benign	Leydig cell tumor	0.00
10	29	Benign	Leydig cell tumor	0.00
11	42	Benign	Leydig cell tumor	0.00
12	31	Benign	Leydig cell tumor	0.00
13	42	Benign	Leydig cell tumor	0.00
14	36	Benign	Leydig cell tumor	0.75
15	69	Benign	Leydig cell tumor	0.00
16	69	Benign	Leydig cell tumor	0.00
17	39	Benign	Leydig cell tumor	0.00
18	39	Benign	Leydig cell tumor	6.96
19	28	Benign	Leydig cell tumor	0.00
20	28	Benign	Leydig cell tumor	0.00
21	34	Benign	Leydig cell tumor	0.00
22	50	Benign	Leydig cell tumor	0.00
23	46	Benign	Leydig cell tumor	0.00
24	51	Benign	Leydig cell tumor	0.99
25	37	Benign	Leydig cell tumor	0.74
26	37	Benign	Leydig cell tumor	0.00
27	49	Benign	Leydig cell tumor	1.82
28	38	Benign	Leydig cell tumor	0.00
29	79	Benign	Leydig cell tumor	0.00
30	32	Benign	Leydig cell tumor	0.00
31	33	Benign	Leydig cell tumor	0.00
32	61	Benign	Leydig cell tumor	0.00
33	64	Benign	Leydig cell tumor	0.43
34	53	Benign	Leydig cell tumor	2.17
35	64	Benign	Leydig cell tumor	0.00
36	40	Benign	Leydig cell tumor	0.00
37	49	Benign	Leydig cell tumor	0.00
38	44	Benign	Leydig cell tumor	0.00
39	50	Benign	Leydig cell tumor	0.00
40	49	Benign	Leydig cell tumor	0.50
41	31	Benign	Sertoli cell tumor	0.00
42	40	Benign	Sertoli cell tumor	0.00
43	62	Benign	Sertoli cell tumor	0.00
44	18	Benign	Sertoli cell tumor	0.00
45	47	Benign	Sertoli cell tumor	0.00
46	48	Benign	Sertoli cell tumor	0.00
47	26	Benign	Sertoli cell tumor	0.00
48	29	Benign	Sertoli cell tumor	0.00
49	37	Benign	Sertoli cell tumor	0.00
50	55	Benign	Sertoli cell tumor	0.00
51	37	Benign	Sertoli cell tumor	0.00
52	41	Benign	Sertoli cell tumor	0.00
53	26	Benign	Sertoli cell tumor	0.00
54	43	Benign	Sertoli cell tumor	2.35
55	18	Benign	Sertoli cell tumor	0.00
56	67	Benign	Sertoli cell tumor	0.00
57	51	Benign	Sertoli cell tumor	0.00
58	49	Benign	Sertoli cell tumor	0.71
59	32	Benign	Granulosa cell tumor	0.00
60	22	Benign	Granulosa cell tumor	0.00
61	21	Benign	Unspecified GST	0.32
62	n.a	Benign	Adenomatoid tumor	0.00
63	29	Benign	Adenomatoid tumor	0.00
64	51	Benign	Adenomatoid tumor	0.81
65	34	Benign	Adenomatoid tumor	0.00
66	34	Benign	Adenomatoid tumor	0.00
67	44	Benign	Adenomatoid tumor	0.00
68	81	Benign	Hemangioma	0.00
69	64	Benign	Hemangioma	0.00
70	74	Malignant	Lymphoma	0.00
71	66	Malignant	Lymphoma	0.00
72	70	Malignant	Lymphoma	0.00
73	n.a	Malignant	Lymphoma	0.00
74	n.a	Malignant	Lymphoma	0.00
75	74	Malignant	Lymphoma	0.00
76	83	Malignant	Lymphoma	0.00
77	89	Malignant	Lymphoma	0.00
78	46	Malignant	Lymphoma	0.00
79	53	Malignant	Lymphoma	0.00
80	69	Malignant	Lymphoma	0.00
81	27	Malignant	Lymphoma	0.00
82	76	Malignant	Lymphoma	0.00
83	62	Malignant	Lymphoma	3.03
84	40	Malignant	Lymphoma	0.00
85	80	Malignant	Lymphoma	8.34
86	76	Malignant	Lymphoma	0.00
87	61	Malignant	Lymphoma	0.00
88	67	Malignant	Lymphoma	0.00
89	61	Malignant	Lymphoma	2.23
90	85	Malignant	Lymphoma	0.00
91	71	Malignant	Lymphoma	2.06
92	68	Malignant	Lymphoma	0.00
93	70	Malignant	Lymphoma	0.00
94	55	Malignant	Lymphoma	25.28
95	52	Malignant	Lymphoma	0.00
96	69	Malignant	Lymphoma	0.00
97	87	Malignant	Lymphoma	4.26
98	77	Malignant	Lymphoma	0.00
99	99	Malignant	Lymphoma	9.19

*RQ* Relative quantity, *GST* gonadal stroma tumor, *n.a.* not available

**Table 2:** Clinical data and relative M371 expression of serum from analysed patients with malignant testicular germ cell tumours

Patient ID	Age (years)	Histology	Tumour size (mm)	Clinical stage	RQ M371
100	29	Seminoma	30	CS3	734.19
101	44	Seminoma	85	CS2	666.29
102	44	Seminoma	26	CS1	24.25
103	40	Seminoma	22	CS1	24.08
104	35	Seminoma	15	CS1	4.02
105	50	Seminoma	48	CS1	238.86
106	56	Seminoma	19	CS1	1.58
107	19	NS (80% Teratoma, 20% YST)	180	CS3	8779.97
108	30	Seminoma	29	CS1	12.21
109	37	Seminoma	68	CS1	374.81
110	36	Seminoma	22	CS1	46.53
111	26	Seminoma	46	CS2	760.08
112	41	Seminoma	24	CS1	8.46
113	36	Seminoma	32	CS1	359.54
114	25	NS (EC)	26	CS2	2574.36
115	21	NS (90% YST, 10% EC)	37	CS1	125.37
116	33	Seminoma	24	CS1	14.52
117	25	NS (EC)	24	CS1	2033.85
118	34	NS (EC)	n.a	CS2	15.22
119	31	NS (EC)	25	CS2	588.13

*CS* Clinical stage, *EC* Embryonal carcinoma, *NS* Non seminoma, *YST* Yolk sac tumor, *RQ* Relative quantity, *n.a.* not available

**Table 3:** Clinical data and relative M371 expression of serum from analyzed tumour-free patients which served as controls

Patient ID	Age (years)	Diagnosis	RQ M371
120	31	Hydrocele	0.00
121	67	Epididymitis	0.00
122	27	Testicular pain	0.00
123	67	Spermatocele	0.00
124	21	Prostatitis	0.00
125	23	Gonadal dysgenesis	0.00
126	49	Infertility	0.33
127	20	Varicocele	0.00
128	33	Testicular pain	0.72
129	41	Epididymitis	0.00
130	40	Infertility	0.00
131	24	Infertility	0.00
132	61	Infertility	0.00
133	55	Infertility	0.00
134	37	Infertility	0.00
135	38	Testicular microlithiasis	0.00
136	45	Infertility	0.00
137	32	Infertility	0.00
138	31	Infertility	0.00
139	79	Epididymitis	0.00
140	40	Azoospermia	0.00
141	38	Chronic testicular pain	0.00
142	70	Chronic testicular pain	0.00
143	24	Chronic testicular pain	0.00
144	54	Epididymitis	0.00
145	39	Epididymitis	2.50
146	42	Epididymitis	0.00
147	74	Orchitis	7.11
148	43	Epididymitis	0.00
149	79	Epididymitis	0.00
150	63	Testicular pain	0.00
151	62	Epididymitis	0.00
152	62	Epididymitis	0.00
153	50	Epididymitis	0.66
154	24	Chronic testicular pain	0.00
155	32	Epididymitis	0.00
156	39	Epididymitis	0.00

*RQ* Relative quantity

All patients gave informed consent. The study had been ethically approved by Ärztekammer Bremen (#301, decisions Jul 08 and Oct 08, 2015). All study activities had been conducted according to the Declaration of Helsinki of the World Medical Association (as amended by the 64th General Assembly, 2013).

### Measurement of serum levels of miRNA

Serum was obtained after centrifugation of whole blood samples. Serum was kept deep-frozen at  − 80° Celsius until shipping to the study laboratory (University of Bremen) where the samples were further kept deep-frozen until final processing.

The measurement of M371 expression levels in serum was performed as previously described (Dieckmann et al. 2019). Briefly, the total RNA was isolated from 200 µL serum using the miRNeasy Mini Kit according to the supplied protocol (Qiagen, Hilden, Germany). For reverse transcription of microRNA into complementary DNA (cDNA), 6 µL total RNA were added to the master mix of the TaqMan Reverse Transcription Kit including specific microRNA stem-loop primers of miR-371a-3p and miR-30b-5p (Applied Biosystems, Darmstadt, Germany). The reaction was performed in the Mastercycler gradient (Eppendorf, Hamburg, Germany) for 30 min at 16 °C, 30 min at 42 °C and 5 min at 85 °C. A preamplification was performed using the cDNA and 1:100 diluted TaqMan Assays for miR-371a-3p (assay ID 002124) and miR-30b-5p (assay ID 000432). MicroRNA expression was finally quantified on 7500 Fast Real-Time PCR System (Applied Biosystems, Darmstadt, Germany) using FAST Start Universal Probe Master (Roche Diagnostics, Mannheim, Germany). Serum levels of M371 were measured relative to endogenous control miR-30b-5p. The final RQ was calculated by means of the 2-ΔΔCT method according to Livak and Schmittgen (2001).

### Statistical methods

The statistical evaluation was carried out with SPSS version 26 (IBM, Armonk, NY, USA). Receiver operating characteristic curves (ROC) were calculated to document the ability of the M371 test to discriminate GCTs from non-germ cell testicular tumours. Spearman correlation was calculated to explore for correlation of RQ values with age of patients. For the analysis of differences between two independent variables, the Mann–Whitney U test was used. Significance was assumed at *p* < 0.05.

## Results

**Fig. 1:**
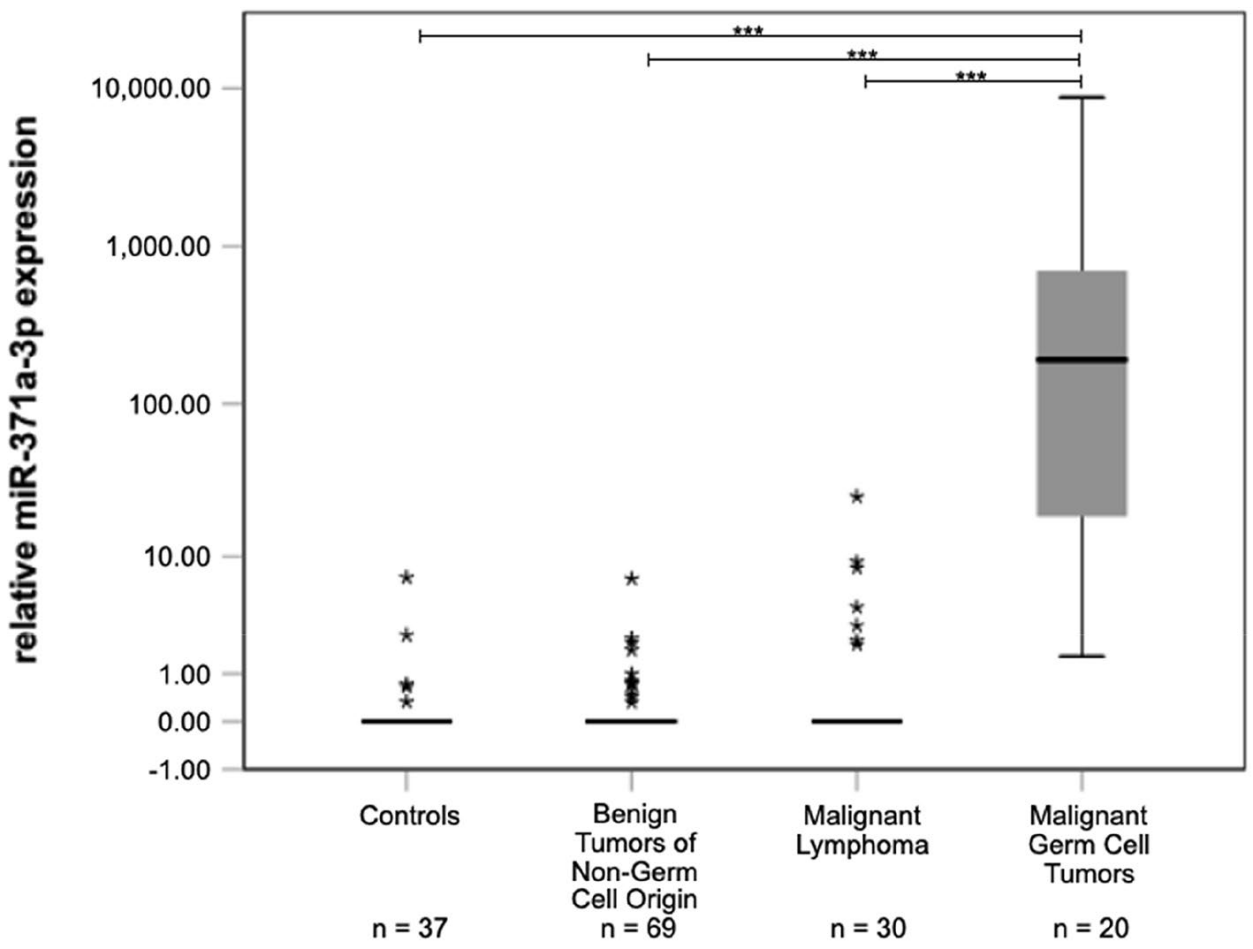
M371 expression in serum of controls and testicular tumours of different histologic subtypes. Boxplots indicate the median relative M371 expressions with interquartile ranges in the various subgroups. Stars represent statistical outliers. The median M371 expression in malignant germ cell tumors is significantly higher than the expressions in controls and in tumours of non-germ cell origin (***p < 0.001). The *y*-axis is shown in a logarithmic scale

In the majority of cases with benign non-germ cell tumours, M371 was undetectable in serum (Fig. 1). The median expression level (relative quantity, RQ) in serum of this subgroup thus is RQ = 0 (interquartile range, IQR = 0) which is not different from controls (RQ = 0; IQR = 0; *p* = 0.659), while TGCT patients had a significantly higher median RQ of 195.54 with an IQR of 717.22–16.91(*p* = 3.21*10–14). Likewise, malignant lymphoma had a median expression level of RQ = 0 (IQR = 0.515–0.000), not different from controls (*p* = 0.206) but significantly lower than the median value of TGCT patients (*p* = 1.97*10–11) (Table 4). Notably, seven patients with malignant lymphoma had somewhat elevated RQ values (outliers in Fig. 1, Table 1), however, these isolated cases did not affect the median RQ value of this subgroup and only three of them ranged above the cut-off level of RQ = 5. Noteworthy, the second highest RQ of 9.19 among the patients with testicular lymphoma was observed in the oldest of these patients. He was 99 years of age and he had bilateral disease. A Spearman correlation revealed that age of the patients does not correlate with RQ (*p* = 0.395). Both of the other outliers with RQ values above the cut-off of RQ = 5, had the rare lymphoma subtype of plasmacytoma, histologically, and both had extended disease with multi-organ affections, clinically.

**Table 4:** Relative expression of M371 in various histological groups of non-germ cell testicular tumours

	Controls	LC tumours	Other GS tumours	Adenomatoid tumours	Hemangioma	Lymphoma	TGCT
*n*	37	40	21	6	2	30	20
RQ median	0.00	0.00	0.00	0.00	0.00	0.00	195.54
RQ minimum	0.00	0.00	0.00	0.00	0.00	0.00	1.58
RQ maximum	7.11	6.96	2.35	0.81	0.00	25.28	8779.97
25th percentile	0.00	0.00	0.00	0.00	0.00	0.00	16.91
75th percentile	0.00	0.00	0.00	0.20	0.00	0.52	717.22

*RQ* Relative quantity, *GS* gonadal stroma tumour, *TGCT* testicular germ cell tumours, *IQR* Interquartile Range

**Fig. 2:**
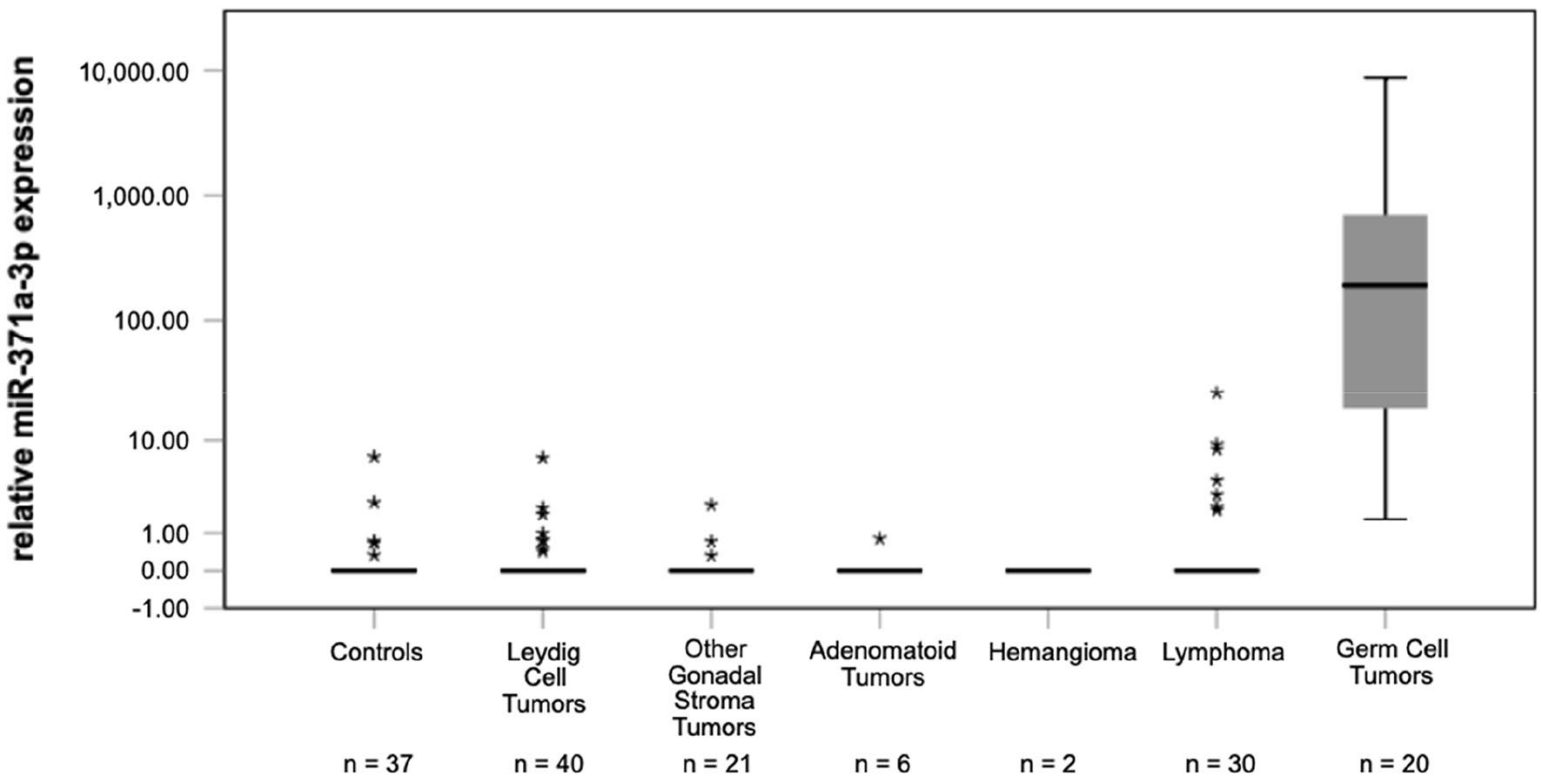
Relative M371 expression in serum of controls, testicular germ cell tumors and in various benign testicular tumours of non-germ origin. Boxplots indicate the median relative M371 expression with interquartile ranges. The subgroup of other gonadal stroma tumours includes Sertoli cell tumours (*n* = 18), Granulosa cell tumours (*n* = 2) and one unspecified gonadal stroma tumour. Statistical outliers are represented by stars. The median M371 expression of germ cell tumours is significantly higher than that of controls and of each of the other subgroups, respectively (***p < 0.001). The y-axis is plotted in a logarithmic scale

Separate analyses of the various histologic subgroups of benign tumours revealed that median M371 levels are zero or close to zero (RQ = 0.00) in serum of patients with any of the histologic subtypes (Fig. 2). The median RQ value of all of the analysed subgroups with testicular tumours of non-germ cell origin is identical with controls, and all subgroups have significantly lower median expression levels of M371 than TGCT (*p* < 0.001). Dieckmann et al. (2017) suggested a RQ of 5.00 to represent an optimal cutoff. In this study within the group of Leydig cell tumours, only one of the eight outliers is located above that cutoff with a RQ of 6.96 but this did not affect the median M371 expression of the group of 0.00 (IQR = 0). Likewise, the three outliers within the group of 21 other gonadal stroma tumours (median RQ = 0; IQR = 0) are close to the median and clearly below the cutoff (Table 4).

**Fig. 3:**
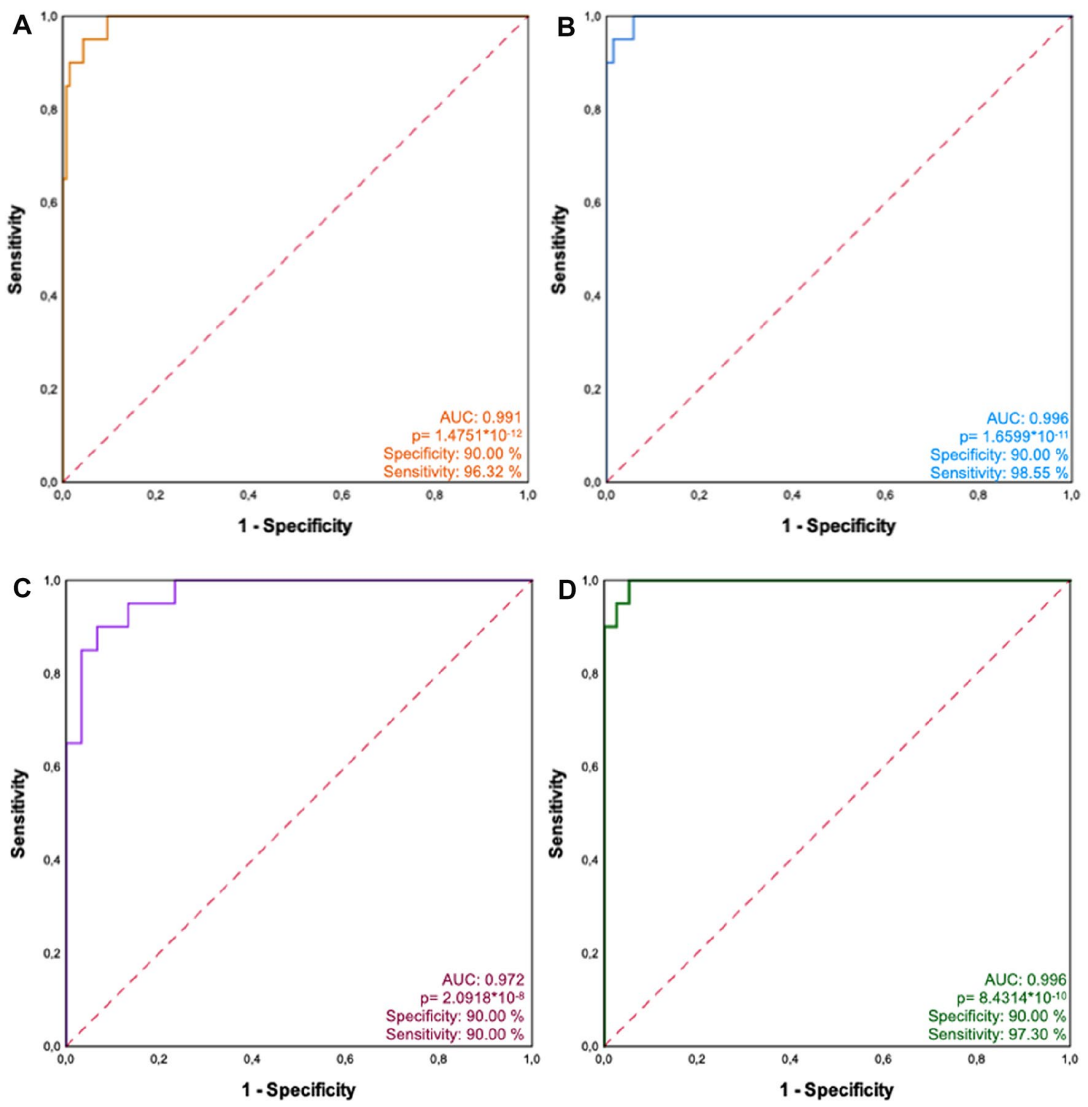
Discriminative ability of M371. ROC curves showing the high sensitivity and specificity of the M371 test for detecting TGCT (*n* = 20) by comparison to (**a**) the entire group of other patients (*n* = 136; AUC, 0.991), **b** patients with benign testicular tumours of non-germ cell origin (*n* = 69; AUC, 0.996), **c** patients with testicular lymphoma (*n* = 30; AUC, 0.972) and **d** control patients (*n* = 37; AUC, 0.996). The specificity of M371 for testicular germ cell tumours is 90% in all calculations. The sensitivity of M371 depends on the histologic subgroup for comparison (**a**–**d**)

The Receiver operating characteristic curves (ROC) based on patients with testicular germ cell tumours (*n* = 20) and the entire group of all other investigated patients with different diseases (*n* = 136) revealed an area under the curve (AUC) of 0.991 (Fig. 3a). With a cutoff of RQ = 5.0, TGCT patients can thus be discriminated from all other histologies with a diagnostic sensitivity of 96.32% and a specificity of 90%. Further ROC analyses revealed an AUC of 0.996 with a sensitivity of 98.55 (Fig. 3b), an AUC of 0.972 with a sensitivity of 90.00% (Fig. 3c), and an AUC of 0.996 with a with a sensitivity of 97.30 (Fig. 3d) when TGCT patients were plotted against patients with benign testicular tumours (*n* = 69), lymphomas (*n* = 30), and control patients (*n* = 37), respectively (all details in composite Fig. 3).

## Discussion

The central result of the present investigation is that non-germ cell neoplasms of the testis do not engender significant elevations of M371 serum levels.

Lower microRNA (miR)-levels in Leydig cell tumours than in TGCTs had already been briefly documented previously (Syring et al. 2015; Dieckmann et al. 2017). However, the present report represents a systematic investigation of a larger number of patients with various types of non-germ cell testicular tumours. The very low or non-expression of M371 in non-germ cell testicular tumours is consistent with the non-expression of this microRNA found in extratesticular malignancies (Spiekermann et al. 2015). Originally, M371 was shown to be characteristic for human embryonic stem cells (Pfaff et al. 2012; Suh et al. 2004). Subsequent investigations found a strong expression of this miR also in germ cell tumours (Belge et al. 2020; Palmer et al. 2010) and it was assumed that the biological similarity of human stem cells and germ cell neoplasms would be the reason for the particular miR profile of testicular tumours (Bezan et al. 2014; Looijenga et al. 2014). In line with that view is the lack of M371 expression in teratoma, because this TGCT subtype represents a much more differentiated, mature neoplasm with almost no features in common with embryonic stem cells (Lobo et al. 2019).

Accordingly, Leydig cell tumours and all other histologic subtypes analysed herein are quite different from embryonic stem cells and from germ cell neoplasms likewise. This dissimilarity regarding morphologic and biologic characteristics is probably the underlying reason for the non-expression of M371 in non-germ cell testicular tumours.

Clinically, the lacking M371 expression of non-germ cell testicular tumours may be a useful tool for preoperative assessment of small testicular neoplasms. It has been shown, that testicular tumours less than 1 cm in size represent benign non-germ cell neoplasms, histologically, in almost two-thirds of the cases (Scandura et al. 2017). Traditionally, every case with suspected testicular neoplasm including all small neoplasms has been considered a candidate for explorative surgery or even orchiectomy (Peckham 1988; Weissbach et al. 1985). With the ever-growing number of incidentally detected cases of small testicular neoplasms, it became clear that many of these cases are of benign nature and would, therefore, require only a conservative surgical approach or even no surgery at all (Dieckmann et al. 2013; Paffenholz et al. 2018). However, approximately 15–20% of all small testicular neoplasms < 1 cm still represent malignant germ cell tumours (Gentile et al. 2020), and therefore the general recommendation for surveillance of small tumours has remained a matter of debate (Ates et al. 2016; Kern et al. 2020; Laclergerie et al. 2018).

The M371 test could aid in clinical decision-making in this setting. The majority of small germ cell tumours < 1 cm of size have shown themselves to instigate elevated M371 serum levels which is particularly true in nonseminomatous tumours. Pure seminomatous tumours have weaker expression of miR, but still 50% of small seminomas show measurable miR-levels (Dieckmann et al. 2019).

Conversely, a negative M371 test found in a patient with a small testicular tumour would thus support the assumption of a benign non-germ cell neoplasm. Accordingly, the negative M371 test might thus allow for clinical decision-making towards a conservative surgical approach or even a surveillance strategy in appropriate cases.

The very low or lacking expression of M371 was consistently found among the various histologic subtypes of non-germ cell testicular tumours analysed herein. Of note, we found somewhat elevated miR-levels in seven patients with malignant lymphoma, but only three of whom had elevations above the cut-off of RQ = 5. Curiously, among these three outliers was the oldest patient aged 99 years who had bilateral testicular lymphoma. He had the second highest RQ value of 9.19. An additional explorative analysis revealed that there is no correlation of RQ values with age in testicular lymphoma. An association of RQ values with tumour load in testicular lymphoma or specific histological subtypes remains conceivable, since two of the outliers had the rare subtype of plasmacytoma and both of whom had extended disease. However, this hypothesis could not be further substantiated in our study because of lacking data on tumour load in the other cases. Currently, there is no clear biological explanation for the number of outliers. At least five outliers were observed among controls, too. Therefore, these somewhat elevated miR levels in a few patients with testicular lymphoma may well reflect the natural scattering of miR-levels and thus most probably represent chance findings.

The present study is certainly not without limitations. Small patient numbers among the histologic subgroups and the retrospective patient accrual are probably the most important limitations. However, as the neoplasms analysed in this study represent truly rare diseases, there is clearly no other way of recruiting such patients for analysis. Thus, the sample sizes appear quite reasonable. A theoretical drawback could be long deep-frozen storage time of serum samples and long shipping times from bedside to laboratory with putative loss of microRNA content. The group of malignant testicular lymphoma was not systematically subdivided, histologically, although several particular subtypes may occur. It remains thus unknown if the outliers in the lymphoma group belong to specific histologic subtypes.

## Conclusions

Non-germ cell tumours of the testis have very low to zero expression of M371 in serum. The present study confirms that the expression of this miR is highly specific for germ cell neoplasms but not for testicular tumours in general. This result might be of value clinically, as in incidentally detected small testicular neoplasms, a negative M371 test could support the diagnosis of a benign non-germ cell tumour and thus facilitate clinical decision-making in favor of conservative surgery or surveillance of small testicular tumours.

## Abbreviations

AFP: Alpha fetoprotein
AUC: Area under the curve
bHCG: Beta human chorionic gonadotropin
LDH: Lactate dehydrogenase
cDNA: Complementary DNA
CS: Clinical Stage
EC: Embryonal carcinoma
GST: Gonadal stroma tumour
IQR: Interquartile range
M371: MicroRNA-371a-3a
miR: MicroRNA
NS: Nonseminoma
qPCR: Quantitative real-PCR
ROC: Receiver operating characteristic
RQ: Relative quantity
SPSS: Statistical package for the social sciences
TGCT: Testicular germ cell tumour
YST: Yolk sac tumour
